# Transcriptomic Insights into the Diversity and Evolution of Myxozoa (Cnidaria, Endocnidozoa) Toxin-like Proteins

**DOI:** 10.3390/md20050291

**Published:** 2022-04-26

**Authors:** Bin Xiao, Qingxiang Guo, Yanhua Zhai, Zemao Gu

**Affiliations:** 1 Department of Aquatic Animal Medicine, College of Fisheries, Huazhong Agricultural University, Wuhan 430070, China; ben.xiaobin@outlook.com (B.X.); qingxiang.guo@outlook.com (Q.G.); zhaiyh@mail.hzau.edu.cn (Y.Z.); 2 Hubei Engineering Technology Research Center for Aquatic Animal Diseases Control and Prevention, Wuhan 430070, China; 3 Engineering Research Center of Green Development for Conventional Aquatic Biological Industry in the Yangtze River Economic Belt, Ministry of Education, Wuhan 430070, China

**Keywords:** venomics, cnidaria, Myxobolidae, phylogenetics, selection analysis, adaptive evolution

## Abstract

Myxozoa is a speciose group of endoparasitic cnidarians that can cause severe ecological and economic effects. Their cnidarian affinity is affirmed by genetic relatedness and the presence of nematocysts, historically called “polar capsules”. Previous studies have revealed the presence of toxin-like proteins in myxozoans; however, the diversity and evolution of venom in Myxozoa are not fully understood. Here, we performed a comparative analysis using the newly sequenced transcriptomes of five Myxobolidae species as well as some public datasets. Toxin mining revealed that myxozoans have lost most of their toxin families, while most species retained Kunitz, M12B, and CRISP, which may play a role in endoparasitism. The venom composition of Endocnidozoa (Myxozoa + *Polypodium*) differs from that of free-living cnidarians and may be influenced by ecological and environmental factors. Phylogenetic analyses showed that toxin families of myxozoans and free-living cnidarians were clustered into different clades. Selection analyses showed that purifying selection was the dominant evolutionary pressure in toxins, while they were still influenced by episodic adaptive selection. This suggests that the potency or specificity of a particular toxin or species might increase. Overall, our findings provide a more comprehensive framework for understanding the diversity and evolution of Myxozoa venoms.

## 1. Introduction

Cnidarians (anthozoans, medusozoans, and endocnidozoans) are among the earliest diverging extant venomous animals [1,2]. Their toxins are produced by the Golgi apparatus of stinging cells (cnidocytes), which are then housed in cells called nematocysts [3]. The venom of medically relevant species (box jellyfish), or easily collected species (sea anemones), has been extensively studied [4]. However, relatively few studies have been conducted within the parasitic cnidarians—Endocnidozoa. Considering that ecological factors have a profound influence on intra-species venom variation [5], this bias will not only compromise our understanding of venom diversity but also limit the exploration of venom function [6].

Myxozoans are obligate endoparasitic cnidarians that form Endocnidozoa with *Polypodium hydriforme* [7,8]. They have a complex life cycle that involves vertebrate and invertebrate hosts [9,10]. Their cnidarian affinity is confirmed by genetic relatedness and the presence of nematocysts known as polar capsules [11]. Unlike their free-living relatives, myxozoans do not use their nematocysts for feeding or defense, but rather discharge the tubules to anchor the spore to the host during infection [12]. It is unclear how this unique life history and the different utilization of nematocysts affect the venom composition of myxozoans.

Advances in omics technologies have enabled the investigation of the toxin components in several myxozoans at transcriptomic or proteomic level. Comparative analysis using the *Kudoa iwatai* genome and transcriptome did not detect venom proteins among the cnidarian-restricted genes [13]. This was further supported by the proteomic analysis of polar capsules isolated from *Ceratonova shasta*, which found minimal evidence of proteins similar to the toxins [14]. These studies suggested a hypothesis that myxozoans might lose their venom during the transition from a free-living lifestyle to parasitism. However, subsequent venomic studies indicated that venom might be retained in myxozoans and used to infect hosts. For instance, transcriptomic analysis of *Myxobolus pendula* identified 49 putative toxin proteins [15]. Venomic analysis of some myxozoans provided evidence for the expression and translation of toxin homologs [6,16]. These studies have conducted pioneering works and provided valuable datasets. However, a comprehensive understanding of venom in the entire Myxozoa group is still lacking. Furthermore, previous studies have not fully explored the evolution of Myxozoa venoms. Unraveling the evolutionary patterns of venom in these dual-host parasitic groups may provide new insights into the mechanisms of venom diversification and molecular evolution in cnidarians.

Myxobolidae is a widespread and diverse component of Myxozoa. With some 1300 species currently described, they represent around 50% of the species diversity of myxozoans [17,18,19]. Certain Myxobolidae species cause emerging diseases. For example, *Myxobolus cerebralis* can cause the well-known whirling disease [20]. Some species of *Myxobolus* and *Thelohanellus* are also pathogenic to their hosts [21,22,23]. These diseases have impacted wild populations of some most iconic fish, resulting in substantial economic loss in aquaculture and fisheries [24]. As a result of these developments, Myxobolidae is now considered to be of ecological, economic, and medical significance, thus representing an excellent candidate for venom study.

Here, we conducted venomic research on newly sequenced transcriptomes of five Myxobolidae species (*Thelohanellus kitauei*, *Myxobolus xiantaoensis*, *Myxobolus ampullicapsulatus*, *Myxobolus turpisrotundus,* and *Myxobolus honghuensis*) and some representative cnidarians. In the present work, we aimed to (i) reveal the distribution patterns of venom in the Myxozoa through a customized bioinformatics pipeline; (ii) compare the venom gene profiles between myxozoans and free-living cnidarians; (iii) explore the evolution of myxozoan venoms using phylogenetic and selection pressure analyses.

## 2. Results

### 2.1. Sequencing and De Novo Assembly of Transcriptome

A total of 67 gigabases (Gb) of compressed raw reads were generated for five myxozoans using paired-end sequencing. The assemblies ranged from 23,995 to 174,559 unigenes with an N50 range from 381 to 1368. Overall completeness was evaluated by CEGMA and BUSCO (Table 1).

### 2.2. Identification of Toxin-like Proteins (TLPs) in Transcriptomes and Genomes

**Figure 1:**
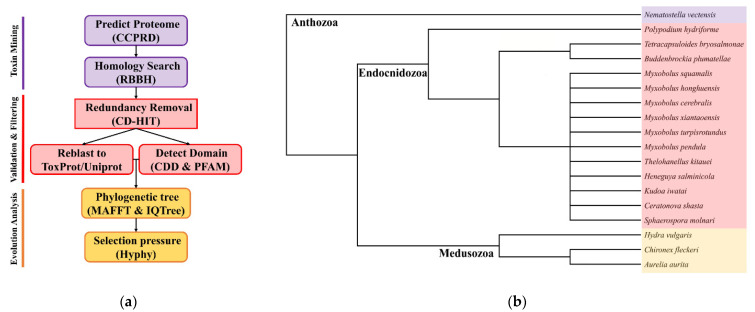
(**a**) The customized bioinformatics pipeline used in this study identified and filtered toxin-like proteins from the sequenced datasets and available omics data; (**b**) cladogram of species used in this study including 14 Endocnidozoa taxa. Adapted from Klompen et al. (2021) and Hartigan et al. (2021) [6,25].

**Figure 2:**
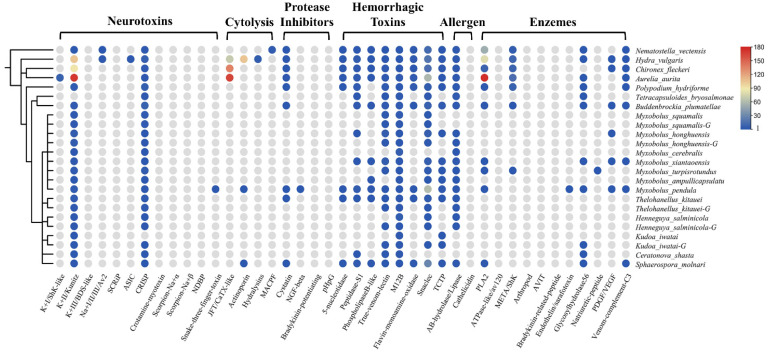
The presence and absence of venom protein families in myxozoans and some cnidarians. A schematic tree of the species used in this project is shown on the left. The circles indicate the presence of a toxin family in that species, and the different colors represent the number of that family. The color gradient on the right corresponds to the numbers. Gray means absent.

Using the customized bioinformatics pipeline depicted in Figure 1a, we found a diverse set of TLPs. Toxins were classified as neurotoxins, cytolysins, protease inhibitors, hemorrhagic toxins, allergens, and enzymes according to their biological functions. The presence and absence of toxin families are shown in Figure 2. We also calculated the proportion of different toxin types in all datasets (Table 2). In general, we found significantly fewer TLPs in myxozoans (range 4 to 88) compared to free-living cnidarians (88, 419, 273, and 611 for *Nematostella vectensis*, *Hydra vulgaris*, *Chironex fleckeri,* and *Aurelia aurita*).

The venom system of cnidarians is mainly composed of three toxin types: enzymes, pore-forming toxins, and neurotoxins [2,26]. As expected, we could not find most of the above toxin families in myxozoans, such as type I and III K^+^ channel inhibitors, any Na^+^ channel inhibitors, SCRIP, ASIC, JFT, hydralysins, MACPF, or ATPase-like toxins. However, we found M12B in all species (*n* = 37), which is classed as a hemorrhagic toxin. Most of the myxozoans, except *Tetracapsuloides bryosalmonae*, possessed the type II K^+^ channel inhibitor Kunitz (*n* = 39). Kunitz is a dual function toxin that inhibits serine protease and blocks potassium channels [27]. We also detected five PLA2 TLPs in *M. xiantaoensis*, *M. turpisrotundus*, *M. pendula*, *Sphaerospora molnari*, and *Buddenbrockia plumatellae*. Interestingly, we found two actinoporin TLPs in *M. pendula* and *S. molnari*, a family of venom proteins usually reported in anthozoans and hydrozoans [2].

Our survey also included toxin families from snakes, scorpions, spiders, cone snails, and insects. The most diverse type was snaclec (*n* = 124) with *M. pendula* and *S. molnari* contributing 62 and 28 respectively. Nearly all species had true venom lectin (*n* = 28) and CRISP (*n* = 39). Interestingly, we found many TCTP in myxozoans, which was an important part of the sea anemone defense system [28]. We also detected snake three-finger toxin (TFT) in *M. pendula*. TFT is a non-enzymatic peptide with multiple biological activities found in all snakes [29].

**Figure 3:**
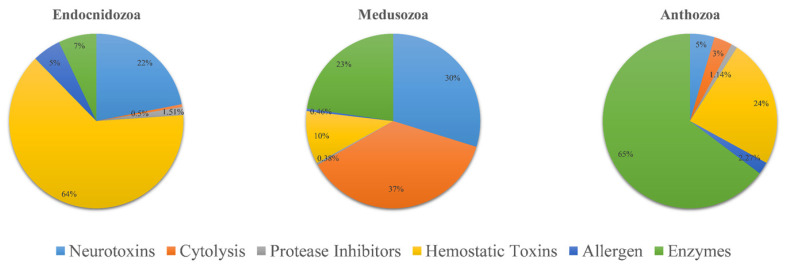
Comparative analysis of putative toxins identified from transcriptomic datasets. Transcripts were identified as coding for potential venoms: Anthozoa (*N. vectensis*, *n* = 88), Medusozoa (*H. vulgaris*, *C. fleckeri*, *A. aurita*, *n* = 1303), and Endocnidozoa (*P. hydriforme* + some myxozoans, *n* = 397).

The venom composition is shown in Figure 3. Overall, the pattern of TLPs in Endocnidozoa was similar. Hemorrhagic toxins were the most diverse family of toxins; they accounted for more than 63% of TLPs. The next most common were neurotoxins (21.9%). Enzymes, allergens, protease inhibitors, and cytolysins were also found in the endocnidozoans, but they accounted for only a small fraction of TLPs. In *N. vectensis*, we found that the most diverse toxin family was enzymes, which constituted more than 64.7% of TLPs. For medusozoans, cytolysins were found to be the most diverse toxin family (36.8%).

Our analysis was mainly based on transcriptomes, but we also added five genomes (*M. squamalis*, *T. kitauei*, *H. salminicola*, *K. iwatai*, and *M. honghuensis* [23,30,31]) to the project to detect the extent of trait differences. When comparing the results of genomic and transcriptomic analyses, we found that most of the species had more TLPs in the transcriptomes, such as *M. squamalis* (11 vs. 9), *M. honghuensis* (19 vs. 12), *T. kitauei* (20 vs. 10), and *H. salminicola* (9 vs. 8). However, there were more TLPs found in the *K. iwatai* genome (*n* = 9 vs. 4 in transcriptome).

### 2.3. Phylogenetic Analysis of Toxin Families

We have developed a phylogenetic framework to reveal the evolutionary patterns and interspecies differences of Myxozoa venoms. Seven toxin families were selected for in-depth analysis, including Kunitz, CRISP, actinoporin, peptidase S1, true venom lectin, M12B and PLA2. These families were selected because they were found in most species or had essential biological functions. These both were frequently studied in the cnidarian literature and other venomous lineages, allowing easier comparison.

#### 2.3.1. Neurotoxins

**Figure 4:**
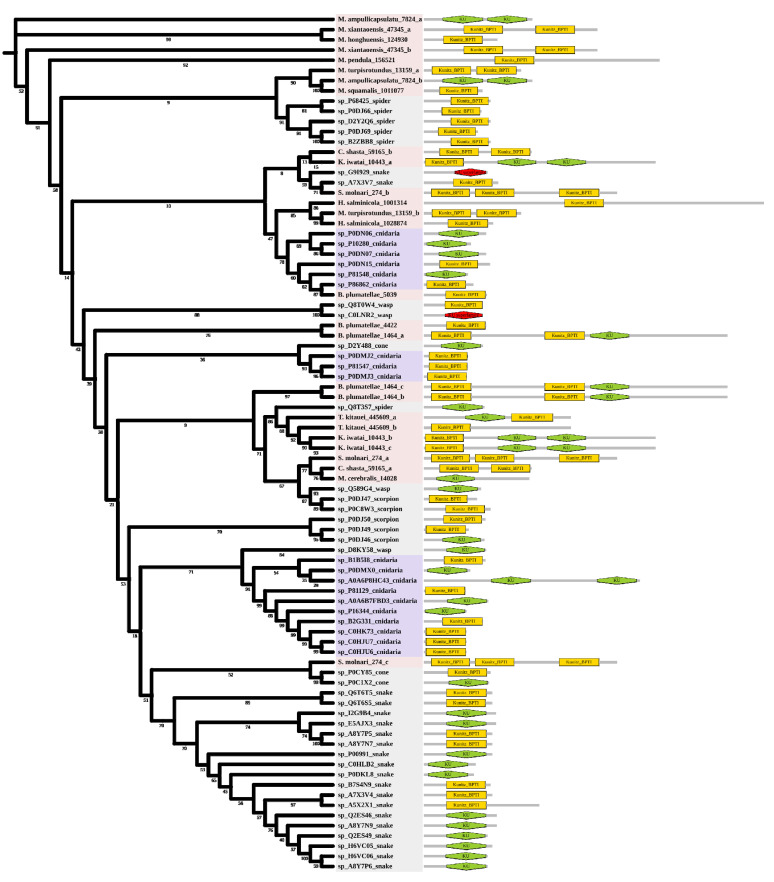
Phylogenetic tree of the Kunitz venom protein family. The maximum likelihood (ML) tree was constructed based on amino acid sequences using IQ-TREE version 2.1.1 software. The numbers below the nodes show bootstrap support values from 1000 replicates. ML phylogram was generated from amino-acid alignments of Kunitz homologs using the auto model. The location and length of conserved domains for each amino acid are shown on the right. Putative sequences outlined in pink, purple, and grey are from myxozoans, free-living cnidarians, and other toxic organisms.

Kunitz is a peptide with a molecular weight of about 6 kDa and usually has a dual function. Six myxozoan clades were supported. Some myxozoan sequences clustered with non-cnidarian species include the sequence of *S. molnari* clustered with the snake (A7X3V7), which is a serine protease inhibitor found in *Philodryas olfersii* [32]. The branch containing *T. kitauei*, *K. iwatai*, and spider (Q8T3S7) was supported by a relatively high bootstrap (86). Q8T3S7 was a putative insecticidal toxin that could inhibit trypsin [33]. We found a sequence of *S. molnari* clustered with cone snails (P0CY85 P0C1X2). They both specifically block voltage-activated potassium channels [34,35]. The free-living cnidarians formed three clades and had little intermixing with myxozoans. In contrast to other venomous taxa, domain duplication existed in both free-living cnidarians and myxozoans. We found two KU domains in *Actinia tenebrosa* (A0A6P8HC43), and three KU/Kunitz-BPTI domains in most myxozoans (Figure 4), which meant the duplication occurred twice in myxozoan at least.

CRISP is a single-polypeptide protein with a molecular weight of 20 to 30 kDa [36]. In reptilian venom, CRISP blocks nucleotide-gated and inhibits potassium channels [37,38]. Two myxozoan clades were supported but did not cluster with other venomous taxa (Supplementary Materials Figure S1).

#### 2.3.2. Enzymes

**Figure 5:**
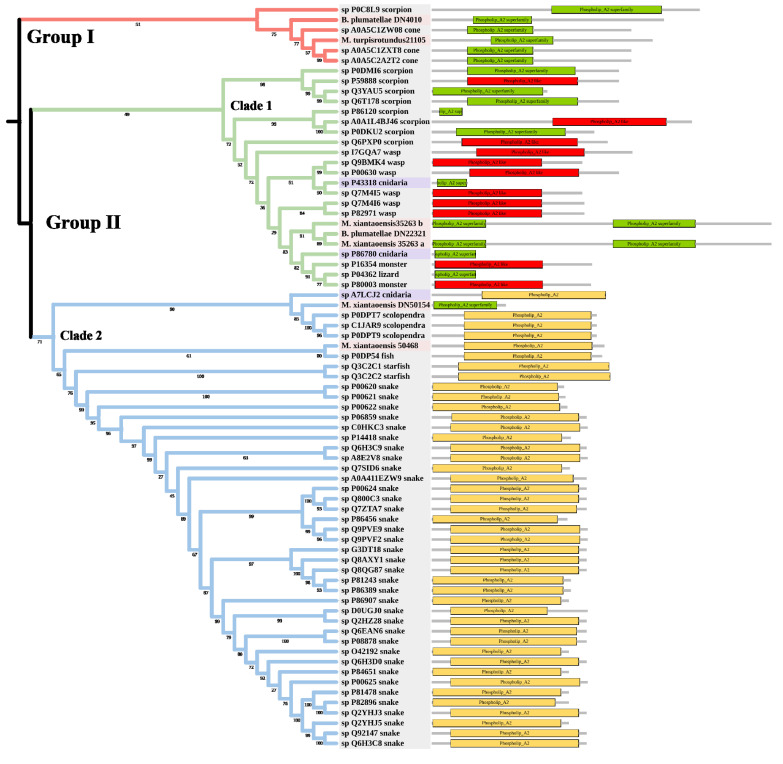
Phylogenetic tree of the PLA2 venom protein family. The maximum likelihood (ML) tree was constructed based on amino acid sequences using IQ-TREE version 2.1.1 software. The numbers below nodes show bootstrap support values from 1000 replicates. ML phylogram was generated from amino-acid alignments of PLA2 homologs using the auto model. The location and length of conserved domains for each amino acid are displayed on the right. Putative sequences outlined in pink, purple, and grey are from myxozoans, free-living cnidarians, and other toxic organisms.

PLA2 has been found in anthozoans, scyphozoans, hydrozoans, and cubozoans [39,40]. The toxic functions of PLA2 in cnidarian venoms include defense, prey digestion, and hemolytic activity [41,42]. The tree was divided into Group I and Group II (Figure 5). Two myxozoan sequences, three cone snail sequences, and one scorpion sequence formed Group I. Group II contained two main clades. Clade 1 included many venomous taxa, such as scorpions and wasps. The three myxozoan sequences were classed into Clade 1. Clade 2 mainly included snakes. There were only two myxozoan sequences in Clade 2: *S. molnari* clustered with scolopendras, and *M. pendula* clustered with a Blenniidae fish *Meiacanthus atrodorsalis*.

#### 2.3.3. Cytolysins

Actinoporins are α-PFTs occurring in anthozoans and hydrozoans, with cardiovascular, respiratory, and cytotoxic effects [43]. We found two actinoporin-like sequences in *M. pendula* and *S. molnari* (Figure S2).

#### 2.3.4. Hemorrhagic Toxins

**Figure 6:**
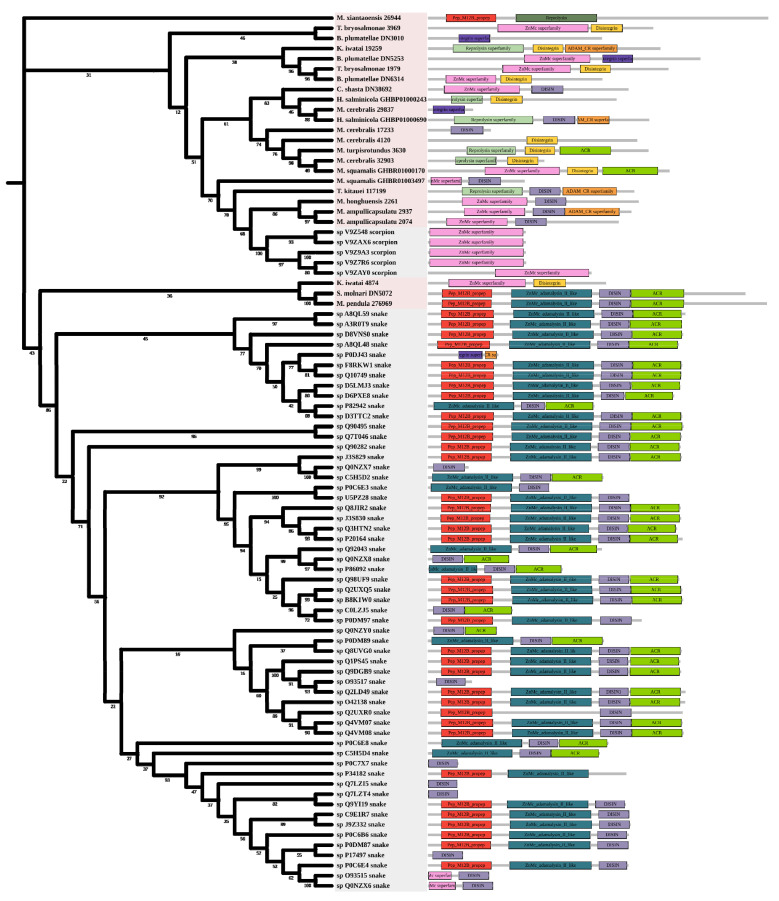
Phylogenetic tree of the M12B venom protein family. The maximum likelihood (ML) tree was constructed based on amino acid sequences using IQ-TREE version 2.1.1 software. The numbers below nodes show bootstrap support values from 1000 replicates. ML phylogram was generated from amino-acid alignments of M12B homologs using the auto model. The location and length of conserved domains for each amino acid are displayed on the right. Putative sequences outlined in pink, purple, and grey are from myxozoans, free-living cnidarians, and other toxic organisms.

Metalloproteinases were mainly found in snakes. Several biological effects are attributed to them including hemorrhage, hypotension, and necrosis [44]. Most myxozoan sequences were clustered in a large group and divided into three clades. A group of *K. iwatai*, *S. molnari*, and *M. pendula* was clustered. Additionally, we detected domain recruitment in the M12B tree (Figure 6).

Peptidase S1 is an enzyme that cleaves peptide bonds in proteins [45]. The phylogenetic tree supported three myxozoan clades (Figure S3). One group was clustered with snakes. While the other was clustered with arthropods. The last group only consisted of *M. pendula* and *S. molnari*, supporting a high bootstrap (100).

True venom lectins bind carbohydrates in snakes [46], exhibiting hemagglutination and platelet aggregation in other species [47]. Four myxozoan clades were supported, most of them belonging to Myxobolidae (Figure S4). Two sequences from *S. molnari* and *M. pendula* were clustered with toadfish (Q66S03) [48].

### 2.4. Selection Pressure Analysis

Considering the obvious differences in phylogeny between free-living cnidarians and myxozoans, we also analyzed the selection pressures to further explore the evolutionary patterns of Myxozoa venoms. Using the codeml program in PAML v4.9 [49] with NSITES = 0, we found the overall dN/dS ratio for Kunitz, M12B and CRISP was consistently 1 (Table 3). The dN/dS ratio is higher for M12B (ω = 0.09904) compared to CRISP (ω = 0.09041) and Kunitz (ω = 0.05767).

Furthermore, we used gene-specific and branch-specific tests implemented through HyPhy [50]. First, we used the Fixed Effects Likelihood (FEL) [51] test to validate the dN/dS ratio. We found that 32 sites in Kunitz, 98 sites in CRISP, and 29 sites in M12B were under purifying selection. Using the Mixed Effects Model of Evolution (MEME) [52] test, we found evidence of episodic individual-sites positive selection across all toxin families. Four sites were found to be evolving under the influence of episodic diversifying selection in M12B. Three sites were found in CRISP and one site in Kunitz. Adaptive branch-site random effects likelihood (aBSREL) [53] found evidence for episodic positive selection across all toxin families except for Kunitz. We detected two branches in CRISP and one branch in M12B. We also used Branch-Site Unrestricted Statistical Test for Episodic Diversification (BUSTED) [54] to detect episodic gene-wide positive selection. The results showed that CRISP had undergone positive selection at least one site on at least one branch. Using the RELAX [55] test, we found the k value for Kunitz and CRISP was 0.88 and 0.99 respectively. Their selection intensification was not significant. While the k value of M12B was 1.07, the test of selection intensity was not significant.

## 3. Discussion

Here, we sequenced and assembled the transcriptomes of five Myxobolidaes species, with BUSCO scores ranging from 57–78%. However, we do not think this is a defect in the data and will not affect the validity of our results. First, the fast evolutionary rates of myxozoans have led to underperformance in both BUSCO and CEGMA [30]. Secondly, the transcriptome sequencing was deep and our samples contained spores from different stages [56].

Our results suggest that the venom composition of endocnidozoans differs from that of anthozoans and medusozoans. Hemorrhagic toxins were the most diverse type of toxin family in endocnidozoans. Anthozoans were rich in enzymes and medusozoans contained more cytolysins and neurotoxins. There are strong indications that various ecological and environmental factors (diet, gender, and geographical distribution) might influence venom composition in animals [57]. We speculate that the use of venom in myxozoans has changed during the transitions to parasitism. They did not use venom for hunting or defense in the same manner as their free-living relatives. A large number of hemorrhagic toxins may be used for host infection or immune escape. Further experimental exploration is needed to understand this question. In addition, we found there were few differences in the types and composition patterns of venom among myxozoans. We speculate that different myxozoans in different living environments may be similar in their utilization of the venom. Future research can expand the scope of species, and combine bioinformatics analysis with functional experiments to explore this issue, which may help explain some interesting biological phenomena in myxozoans.

We noted some differences in venom-mining results at the transcriptomic and genomic levels. Overall, we found more TLPs in transcriptomes, suggesting that gene duplication may occur in some toxin families. Some toxin families were found in transcriptomes but not in genomes, we speculate that this may be caused by alternative splicing, which has been proved to influence transcriptional and post-transcriptional regulation of venom genes [58]. Considering that our proposed set of toxin-like proteins is seen as a first step in exploring the diversity and evolution of an understudied group, future research can build on this foundation to examine the effects of alternative splicing on the myxozoan venom, and determine the mechanisms of diversification. Meanwhile, some toxin families were only found in the genome. This can be a result of selective loss or “decommissioning” of protein families from the venom [1]. We have detected many TLPs with different functions in myxozoans, but the protein expression level of TLPs may be inconsistent with the hints in genomes and transcriptomes. For example, it has been shown in the mixed myxosporean that even when more serine peptidase sequences are present in the transcriptome, the expression of neurotoxins is greater overall at the proteomic level [6]. Thus, future quantitative gene expression and proteomic studies are needed to provide a holistic understanding of myxozoan venoms.

Compared to free-living cnidarians, myxozoans have lost most of their toxin families. However, we can identify a small set of toxin families common to almost all myxozoans, such as Kunitz, CRISP, and M12B. These “core” toxin families may consist of proteins with an ancient and conserved role in nematocyst function [1], which may play a key role in the adaptive evolution of myxozoans. Previous studies support the idea that these protein families are closely related to parasitism. For example, Kunitz is identified in the transcriptome of *Echinococcus granulosus* and has been shown to interfere with host physiological processes during the initial stages of infection [59]. In addition, CRISP is thought to play an important role in the development and parasite adaptation of cestodes [60]. Metalloproteinases have been identified in ectoparasitic wasps with abilities to manipulate host development [61]. To gain a better understanding of these “core” toxin families, we focused on their evolutionary history in our subsequent studies.

Kunitz-type venom is present in proteins from most venomous taxa and plays an important role in envenomation [62]. In sea anemones, Kunitz is classified as a type II K^+^ channel inhibitor. It protects the organisms from endogenous proteases, causes paralysis in prey, and defends against enemies [63,64]. Molecular phylogenetic analysis of Kunitz showed that myxozoans and free-living cnidarians are clustered into distinct clades. Some myxozoan sequences are grouped with other venomous animals. Our results support the hypothesis proposed by Hartigan et al. (2021) that the toxins of endoparasitic cnidarians are more divergent from homologs in free-living relatives [6]. This may be caused by the potential convergent evolution of venoms in diverse animal groups. Interestingly, we found that the Kunitz conserved domain is duplicated in myxozoans and free-living cnidarians, although not in other toxic organisms. Conserved domains represent the basic evolutionary units that form proteins. Domain duplication and reorganization are the most important forces driving protein evolution and increasing proteome complexity [65]. We also note that domain duplication differs in the two groups. Duplication occurred twice in myxozoans and only once in free-living cnidarians. We hypothesize that the Kunitz domain was first duplicated in their common ancestor, and there is a separate duplication for myxozoans after the divergence of endoparasitism and free-living. Further experiments are needed to explore the effect of duplication on the function of Kunitz.

Metalloproteases are important venom components of terrestrial animals [66]. They have also been detected in jellyfishes and sea urchins [67,68,69] with gelatinolytic, caseinolytic, and fibrinolytic activity [49]. The toxicity of CRISP in cnidarians is not fully understood, but it has been detected in anthozoans, scyphozoans, and hydrozoans [1]. M12B and CRISP shared similar phylogenetic results. Most of the myxozoans did not cluster with other venomous taxa. We detected domain recruitment in M12B and domain divergence in CRISP. In many venomous taxa, toxins evolve through a combination of gene recruitment, duplication, and neofunctionalization, leading to extended families of closely related yet often functionally distinct toxin isoforms [70]. In that case, myxozoan may have evolved new functions of M12B and CRISP to support the endoparasitic lifestyles.

The evolution of venom is usually accompanied by strong evidence of accelerated evolution and positive selection [71]. This is especially true in recently diverged venomous clades, such as snakes, scorpions, spiders, and cone snails [72,73,74,75]. In contrast, venom evolution is dominated by purifying selection in ancient venomous lineages (e.g., cnidarians, coleoids, and arthropods) [76]. In the present work, we found that the Myxozoa venoms were under purifying selection, which is consistent with Cnidaria [77]. Why do the evolutionary patterns of cnidarian venoms differ from that of other venomous organisms? This may be because venom production in cnidarians is associated with the development of nematocysts. This will pose genomic constraints on the evolution of venom in cnidarians [1]. However, we detected episodic positive selection on sites or branches in myxozoan toxin families using MEME and aBSREL, which suggests that weak diversity selection was involved in Myxozoa venoms. We also interpret this discrete positive selection as concomitant with increased potency and/or specificity of a particular toxin or species. Overall, these patterns suggest that myxozoan venoms have undergone a varied history of selection pressures, including widespread purifying selection and a few instances of gene-specific or lineage-specific episodic positive selection.

Collectively, we provide a comprehensive view of Myxozoa venoms. We detected relatively few toxin-like proteins in myxozoans, suggesting that the reduction in myxozoan genome size includes reduced toxin diversity [6]. The phylogenetics and selection pressure results reflect differences in venoms between myxozoans and free-living cnidarians, suggesting that toxins might have evolved to adapt to parasitic lifestyles. The set of toxin-like proteins we present is viewed as an initial step into exploring diversity and evolution within a poorly studied group of cnidarians. These new data will be used to further explore the diversification and molecular evolution of toxins encoded by these fascinating and ancient animals.

## 4. Materials and Methods

### 4.1. Collecting Sample

*M. honghuensis* was collected from infected allogynogenetic gibel carp *Carassius auratus gibelio* in Zoumaling Farm, Hubei Province, China on 29 July 2015. *M. ampullicapsulatus* was collected from infected allogynogenetic gibel carp *C. auratus gibelio* in Datonghu lake, Hubei Province, China on 12 May 2016. *M. xiantaoensis* was collected from infected yellow catfish *Tachysurus fulvidraco* in Xiantao, Hubei Province, China on 23 April 2016. *M. turpisrotundus* was collected from infected allogynogenetic gibel carp *C. auratus gibelio* in Wuhan, Hubei Province, China on 16 November 2015. *T. kitauei* was collected from infected common carp *Cyprinus carpio* in Liuerbao Town, Shenyang Province, China on 11 August 2015.

Fish were held on ice before being killed with an overdose of MS-222 (Sigma-Aldrich, Co., Ltd., St. Louis, MO, USA). From each fish, tissue containing one large cyst was homogenized by a manual glass tissue grinder and suspended in 0.1 M phosphate-buffered saline (PBS) with a pH 7.2 and then filtered through cotton gauze. Myxospores were separated from the filtrate by sucrose gradient centrifugation and Percoll gradient centrifugation, in turn. They were washed several times with distilled water and then examined microscopically to verify purity and identity. Purified myxospores were either placed into RNAlater (Sigma), frozen in liquid nitrogen, and finally stored at −80 °C, or immediately sent to nematocyst isolation as described below. Myxozoan identification was performed based on morphology and 18S sequencing [78,79]. Maintenance and care of experimental animals complied with the National Institutes of Health Guide for the care and use of laboratory animals [80] and was approved by the animal care and use committee of Huazhong Agricultural University, China (HZAUFI-2015-011).

### 4.2. Next-Generation Sequencing and Assembly

Total RNA was extracted from frozen preserved specimens using TRIzol 550 (Invitrogen, Carlsbad, CA, USA). The purity and integrity of RNA were assessed using NanoPhotometer^®^ spectrophotometer (IMPLEN, Westlake Village, CA, USA), and RNA Nano 6000 Assay Kit of the Agilent Bioanalyzer 2100 system (Agilent Technologies, Santa Clara, CA, USA) respectively. Libraries were prepared from purified mRNA using NEBNext^®^ Ultra RNA kit (New England Biolab (NEB), Ipswich, MA, USA) following the manufacturer’s recommendations and sequenced as 2 × 125 paired-end (PE) runs with the Illumina HiSeq 2500 for *M. honghuensis* and *T. kitauei*; as 2 × 150 paired-end runs with the Illumina HiSeq 4000 for *M. ampullicapsulatus*, *M. turpisrotundus*, and *M. xiantaoensis*. Raw sequence data were cleaned and trimmed by removing adaptor and low-quality reads using Trimmomatic v0.33 [81]. Filtered reads were de novo assembled through Trinity v2.6.6 [82] and clustered using CD-HIT v4.6.8 [83] and TGICL v2.1 [84]. BUSCO v4.0 [85] and CEGMA [86] were used to evaluate the completeness of assemblies.

### 4.3. Data Decontamination and Construction Predict Proteomes

Here we used our recently developed method to construct a clean, efficient, and comprehensive protein reference that we denote as the customized comprehensive proteomic reference database (CCPRD) (for details, see [56]). Briefly, nonredundant host databases containing proteins or nucleotide sequences from the host and nonredundant closely related databases containing proteins or nucleotide sequences from species closely related to myxozoans were constructed. These four databases were then blasted against the transcriptome assemblies using TBLASTN or TBLASTX in BLAST+ v2.4.0 [87] (e-value 1 × 10^−5^). Only transcriptome sequences that exclusively matched host databases were removed. The retained transcriptome sequences were further blasted against a bacterial database using BLASTX (e-value 1 × 10^−10^). The positive hits were blasted to the NCBI nonredundant protein database (NR) using BLASTX (e-value 1 × 10^−5^). Only sequences that were annotated as “bacteria” were removed. For assessment and visualization of contamination in genomes, taxonomic assignment of each contig was carried out using Blobtools v1.0 [88]. Sequences strongly matched to Chordata and Proteobacteria were excluded. The decontamination process was carried out conservatively to prevent possible over-decontamination that could result in a loss of a large portion of actually expressed genes and proteins.

For RNA-seq data, coding sequences were firstly predicted by TransDecoder [89] and GeneMarkS-T [90]. Next, transcriptome homology-based predictions were c by a customized Perl script Hercules (https://github.com/qingxiangguo/hercules, accessed on 5 April 2020). Furthermore, those transcripts that were translated neither by de novo nor homology-based method were translated into amino acid sequences using the Transeq script from the EMBOSS [91]. Finally, all the proteins (30 amino acids) resulting from genomic and RNA-seq data were processed by CD-HIT with a threshold of 100%, to collapse the group into a nonredundant data set, leading to the final predicted proteomes. Besides the data we sequenced, 18 publicly available transcriptomes and genomes were used in this project (for details, see Table S1).

### 4.4. Identification Toxin in Predict Proteomes

We used a pipeline aimed at conservatively identifying toxins: a group of toxin sequences from cnidarian and other venomous animals was generated as seed for reciprocal best BLAST hits (RBBH) analysis to predict proteomes (e-value 1 × 10^−5^). All the candidate toxin proteins identified by RBBH were validated by blasting against the Tox-Prot UniProtKB/Swiss-Prot [92] and only proteins with the best match to toxins were retained (e-value 1 × 10^−5^). PFAM annotation by HMMER [93] was conducted for those putative toxins. They were considered positive only if the presence of the toxin motif or family domain (partial or complete) could be confirmed in protein sequences. Results were manually curated to confirm that toxin-like sequences matched the detected venom domain from PFAM. CDD annotations were added (See Table S2 for detailed results). The results were visualized using Microsoft Excel, and final figures were constructed in Inkscape v1.0.

### 4.5. Alignment and Phylogenetic Analysis

We used TBtools v1.098669 [94] to extract the domain of toxin protein sequences for conducting phylogenetic trees. Other sequences were obtained from VenomZone (https://venomzone.expasy.org, accessed on 10 May 2020). Retrieved amino acid sequences were aligned with MAFFT v7.205 [95] by the L-INS-i method. Multiple alignments were trimmed using Gblocks v0.91b [96]. Phylogenetic trees were constructed using a maximum likelihood (ML) approach by IQ-TREE v2.1.1 [97] and node support was obtained with 1000 ultrafast bootstrap. Searches with multiple 500 initial parsimony trees were performed. Bootstrap support values were assigned on the best maximum likelihood tree branches. The tree was collapsed and formatted using iTOL v4 [98].

### 4.6. Selection Analysis

We first extracted the coding sequences of the different toxin families using TBLASTN, and then used MEGA-X [99] for sequence alignment and stop codon movement. Codon alignments were obtained by matching the aligned amino acids to their DNA sequences using pal2nal v14 [100] on the online server. The overall synonymous to nonsynonymous substitutions (dN/dS) ratio values were evaluated using codeml. Then, the package HyPhy was used to conduct several positive selection analyses (FEL, MEME, Absrel, BUSTED, and RELAX). Tree topologies were modified to be consistent with the ML tree for all test runs through Datamonkey [101]. Values of dN/dS 0, =0, and 0 indicate negative selection, neutral evolution, and positive selection, respectively.

## Figures and Tables

**Table 1:** Sequencing and assembly summary for five Myxobolidae transcriptomes.

Species	Contigs	Unigene	Mean Length	N50	CEGMA	BUSCO
*T. kitauei*	90,775	88,081	590	1118	67.3%	57.3%
*M. xiantaoensis*	98,837	66,662	844	1368	77.8%	78.4%
*M. ampullicapsulatus*	276,231	174,559	380	381	71.8%	64.7%
*M. turpisrotundus*	171,878	153,649	453	759	62.1%	57.3%
*M. honghuensis*	27,502	23,995	6013	857	59.6%	59.6%

**Table 2:** Composition of venom proteins identified in myxozoans and some cnidarians.

Class/Toxins	Neurotoxins	Cytolysins	Protease Inhibitors	Hemorrhagic Toxins	Allergen	Enzymes	ALL
*N. vctensis*	4/4.5%	3/3.4%	1/1.1%	21/23.9%	2/2.3%	57/64.8%	88
*H. vulgaris*	115/27.4%	179/42.7%	2/0.5%	31/7.4%	2/0.5%	90/21.5%	419
*C. fleckeri*	93/34.1%	136/49.8%	1/0.4%	25/9.2%	2/0.7%	16/5.9%	273
*A. aurita*	180/29.5%	165/27.0%	2/0.3%	72/11.8%	2/0.3%	190/31.1%	611
*P. hydriforme*	8/22.2%	0/0.0%	1/2.8%	16/44.4%	1/2.8%	10/27.8%	36
*T. bryosalmonae*	2/16.7%	0/0.0%	0/0.0%	6/50.0%	3/25.0%	1/8.3%	12
*B. plumatellae*	10/27.0%	0/0.0%	1/2.7%	20/54.1%	2/5.4%	4/10.8%	37
*M. squamalis*	5/45.5%	0/0.0%	0/0.0%	6/54.5%	0/0.0%	0/0.0%	11
*M. squamalis-G* ^1^	5/55.6%	0/0.0%	0/0.0%	4/44.4%	0/0.0%	0/0.0%	9
*M. honghuensis*	5/26.3%	0/0.0%	0/0.0%	13/68.4%	1/5.3%	0/0.0%	19
*M. honghuensis-G* ^1^	6/50.0%	0/0.0%	0/0.0%	5/41.7%	1/8.3%	0/0.0%	12
*M. cerebralis*	2/25.0%	0/0.0%	0/0.0%	5/62.5%	1/12.5%	0/0.0%	8
*M. xiantaoensis*	3/14.3%	0/0.0%	0/0.0%	14/66.7%	2/9.5%	2/9.5%	21
*M. turpisrotundus*	7/38.9%	0/0.0%	0/0.0%	7/38.9%	1/5.6%	3/16.7%	18
*M. ampullicapsulatu*	2/22.2%	0/0.0%	0/0.0%	6/66.7%	1/11.1%	0/0.0%	9
*M. pendula*	6/6.8%	1/1.1%	2/2.3%	74/84.1%	2/2.3%	3/3.4%	88
*T. kitauei*	6/30.0%	0/0.0%	1/5.0%	12/60.0%	1/5.0%	0/0.0%	20
*T. kitauei-G* ^1^	5/50.0%	0/0.0%	0/0.0%	4/40.0%	1/10.0%	0/0.0%	10
*H. salminicola*	3/33.3%	0/0.0%	0/0.0%	5/55.6%	1/11.1%	0/0.0%	9
*H. salminicola-G* ^1^	4/50.0%	0/0.0%	0/0.0%	3/37.5%	1/12.5%	0/0.0%	8
*K. iwatai*	1/25.0%	0/0.0%	0/0.0%	3/75.0%	0/0.0%	0/0.0%	4
*K. iwatai-G* ^1^	2/22.2%	0/0.0%	0/0.0%	6/66.7%	0/0.0%	1/11.1%	9
*C. shasta*	2/28.6%	0/0.0%	0/0.0%	4/57.1%	0/0.0%	1/14.3%	7
*S. molnari*	3/6.0%	1/2.0%	1/2.0%	40/80.0%	2/4.0%	3/6.0%	50

^1^ Represents the genome.

**Table 3:** Results of selection pressure analysis for toxin families.

Method/Family	Kunitz	CRISP	M12B
Codeml	0.05767	0.09041	0.09904
FEL	−32	−98	−29
MEME	1	3	4
aBSREL	0	2	1
BUSTED	found no evidence	found evidence	found no evidence
RELAX	K 1	K 1	K 1

## Data Availability

Data generated and analyzed during this study are included in the published article, its additional files, and publicly available repositories. The raw reads of *M. honghuensis* transcriptome sequencing and PacBio genome sequencing have been deposited at the NCBI Short Read Archive (SRA) with the Bioproject accession numbers PRJNA779260, PRJNA778632, and PRJNA779846. The raw reads of other myxozoan transcriptome sequencing were also deposited under the Bioproject accession numbers in GenBank: *M. ampullicapsulatus* (PRJNA786097), *M. turpisrotundus* (PRJNA786098), *M. xiantaoensis* (PRJNA786274), *T. kitauei* (PRJNA786278, Illumina Hiseq 2500), and *T. kitauei* (PRJNA786276, Illumina Hiseq 4000).

## Supplementary Materials

The following supporting information can be downloaded at https://www.mdpi.com/article/10.3390/md20050291/s1. Figure S1: Phylogenetic tree of the CRISP venom protein family, Figure S2: Phylogenetic tree of the actinoporin venom protein family, Figure S3: Phylogenetic tree of the peptidase S1 venom protein family, Figure S4: Phylogenetic tree of the true venom lectin venom protein family, Table S1: Summary of transcriptomes and genomes used in this project, Table S2: Annotation table for putative toxin-like proteins identified for five Myxobolidae species, File S1: Toxin families used as seeds for RBBH in this project.
